# Candidate microRNA biomarkers in human epithelial ovarian cancer: systematic review profiling studies and experimental validation

**DOI:** 10.1186/1475-2867-13-86

**Published:** 2013-08-27

**Authors:** Ying Chen, Lei Zhang, Quan Hao

**Affiliations:** 1 Department of Gynecologic Oncology, Tianjin Medical University Cancer Institute and Hospital, Huanhuxi Road, Hexi District, Tianjin, 300060, China; 2 Key Laboratory of Cancer Prevention and Therapy, Tianjin, China; 3 National Clinical Research Centre of Cancer, Tianjin, China

**Keywords:** Ovarian cancer, MicroRNA, Biomarker

## Abstract

Despite advances in detection and therapy, epithelial ovarian cancer (EOC) still represents the most lethal gynecologic malignancy in women worldwide. The high mortality of EOC is mainly due to late-stage diagnosis for more than 70% of patients. There is an urgent need to search for specific and sensitive biomarkers for early diagnosis of EOC. Recently, the cumulative data indicated an essential role for microRNA (miRNA), a class of small non-coding RNAs targeting multiple mRNAs and triggering translation repression and/or RNA degradation, in ovarian caner carcinogenesis and progression. Here, we reviewed the published miRNA expression profiling studies that compared the miRNA expression profiles between EOC tissues or cell lines and normal ovarian tissues or benign ovarian tumor or human primary cultured ovarian surface epithelial cells. A miRNA ranking system that takes the number of comparisons in agreement and direction of differential expression into the consideration was devised and used. Finally, five promising differentially miRNAs (miR-200a, miR-100, miR-141, miR-200b, and miR-200c) were reported with the consistent direction in four or more studies. MiR-200a, miR-200b, miR-200c, and miR-141, all of them belong to miR-200 family, were reported with consistently up-regulated in at least 4 studies, whereas miR-100 was reported with down-regulated in 4 studies. Furthermore, we validated these miRNAs in a clinical setting using qRT-PCR and their dysregulations in EOC tissues confirmed the findings. Conclusively, the five most consistently expressed miRNAs might provide some clues of the potential biomarkers in EOC. Further mechanistic and precise validation studies are needed for their clinical significances and roles in the progression of EOC.

## Introduction

Epithelial ovarian cancer (EOC, referred to as ovarian cancer in this review) is the fifth leading cause of cancer death in women and the most lethal gynecologic malignancy in the world [1]. Despite recent advances in chemotherapeutic treatments that have improved the initial responses, the 5-year survival rate for women with advanced stage ovarian cancer is only about 30% after initial diagnosis [2]. Pessimistically, more than 70% of EOC patients appeared the first specific symptoms and are diagnosed only during advanced disease stage [3]. Although various screening methods or specific markers for early detection of EOC are available, their diagnostic values are limited due to lack of sensitivity, high costs and or inconvenience. Thus, there is an urgent need to identify new diagnostic and prognostic biomarkers for EOC.

MicroRNAs (miRNAs) are a class of small (19 to 25 nt), non-coding, highly stable RNAs that regulate mRNA and protein expression. Several studies have indicated that miRNAs have been involved in regulating various biological processes, such as cellular differentiation, proliferation, angiogenesis, metabolism and cancer development [4-6]. Microarray-based miRNA profiling assays attracted more attention because they constitute the efficient methodology to screen in parallel for the expression of hundreds of miRNAs through extensive sample collections. As we know, with the aim at identifying new biomarkers of ovarian cancer, many investigators have carried out miRNAs expression profiling studies in cell lines, tissue or serum samples [7-17]. Notably, dozens of miRNAs are identified to be differentially expressed and can be either up- or down- regulated, depending on their target downstream genes, although only a small fraction of them may actually be of clinical utility as diagnostic/prognostic biomarkers or therapeutic targets.

However, there had been inconsistency or discrepancy in the identified differentially expressed miRNAs among the different profiling studies, which give rise to identify the most consistently reported differentially expressed miRNAs in multiple independent studies necessarily. Although the identification of intersections of the cancer-related genes based on a large number of gene profiling studies has become increasingly popular, there has been no study investigating the intersections of cancer-related miRNAs based on a number of miRNA expression profiling studies in ovarian cancer.

Thus, we summarized this systematic review to determine the differentially expressed up- and down- regulated miRNAs that were consistently reported in different independent miRNA expression profiling studies in EOC. Furthermore, the most consistently expressed miRNAs were validated in a clinical setting.

## Material and methods

### Literatute selection

To identify all relevant literature, we searched PubMed for ovarian cancer miRNA expression profiling studies published between January 2002 and December 2012, by means of the MeSH terms: ‘ovarian neoplasms’ and ‘microRNAs’ in combination with the keyword ‘profiling’ and ‘humans’. Eligible studies had to meet the following criteria: ① they were miRNA expression profiling studies in ovarian cancer patients or ovarian cancer cell lines; ② they used tissue samples obtained from surgically removed EOC specimens or epithelial ovarian cancer cell lines and benign ovarian tumor or normal ovarian tissues or human primary cultured ovarian surface epithelial cells for comparison; ③ use of miRNA microarray methods; ④ reporting of cut-off criteria of differentially expressed miRNAs; ⑤ validation method and validation sample set reported; and ⑥ they were published as full articles in English only. Therefore, the miRNA profiling studies using the serum of ovarian cancer patients, or using different miRNA technologies were excluded. Review articles and studies comparing miRNA expression profiles in EOC with cisplatin-sensitive and cisplatin-resistant or refractory were also excluded.

### Data abstraction

Two investigators (YC and LZ) independently evaluated and extracted the data with the standard protocol and with all the discrepancies resolved by a third investigator (QH). From the full text and corresponding supplement information, the following eligibility items were collected and recorded for each study: author, journal and year of publication, location of study, selection and characteristics of recruited ovarian cancer patients, platform of miRNA expression profiling, author defined cut-off criteria of statistically differentially expressed miRNAs, the list of up- and down-regulated miRNA features, and the frequency of different miRNAs reported among these eligible literatures.

### Ranking

Each of published miRNA expression profiling studies comparing miRNA expression between EOC or ovarian cancer cell lines and benign ovarian tumor or normal ovarian tissues or human primary cultured ovarian surface epithelial cells resulted in a list of differentially expressed miRNAs. These miRNAs were ranked according to several criteria, which were similar to those in Griffith’s [18] and Chan’s [19] studies in the following order of importance: ① the miRNA was consistently reported as differentially expressed; ② the consistently reported differentially expressed miRNA was also in a consistent direction of change; ③ the frequency of the miRNA was reported in the miRNA ecpression profiling studies.

### Validation of the most consistently differentially expressed miRNAs using quantitative real-time PCR

To validate the microarray results, ten fresh EOC specimens were collected from women during primary surgery and prior to the initiation of adjuvant therapy at the Department of Gynecologic Oncology, Tianjin Medical University Cancer Institute and Hospital. Ten normal ovarian tissues were obtained from women who underwent surgery for benign or malignant gynecological diseases other than ovarian carcinoma at the same department. Total RNA was extracted using the Qiagen RNeasy Kit (QIAGEN GmbH, Germany) according to the manufacturer’s instructions. First-strand complementary DNA (cDNA) was synthesized from 2 μg of tatal RNA using an oligo-dT primer and superscript II reverse transcriptase (Invitrogen). Then, quantification was performed by real-time PCR, using SYBRRPremix Ex Taq TM (TakaRa) for the most consistently differentially expressed miRNAs. The primers for U6 were obtained from TakaRa. PCR was performed in a real-time PCR system (BIO-RAD) as follows: 95°C for 3 min followed by 35 cycles of 95°C for 5 sec, 60°C for 20 sec and 72°C for 30 sec and then 94°C for 1 min, 60°C for 1 min, with addition of a cycle for every 0.5°C. Expression values were normalized to those for U6. Relative fold changes of miRNA expression were calculated by the ΔΔCT method, and the values were expressed as 2^-ΔΔCT^.

### Statistical analysis and ethics statement

Statistical analyses were performed by GraphPad Prism Software (GraphPAD Prism Software, Version 5.01, GraphPad, San Diego, CA). Statistical significance was defined as *P<0.05.* All human specimens were obtained with the written informed consent of participants in accordance with the requirements of the Research Ethics Committee of Tianjin Medical University Cancer Institute and Hospital, China.

## Results

### Independent studies for data extraction

In total, 80 studies were searched using PubMed. Following the strict inclusion and exclusion criteria, only 8 independent studies were included in the systematic analysis. The characteristics of these studies are listed in Table 1.

**Table 1:** Eight microRNA expression profiling studies included in the systematic review

**Reference**	**Year**	**Platform**	**No. of differential miRNA**	**Up-regulated**	**Down-regulated**
Kim et al. [7]	2010	MiRNA microarray (human-miRNA-V1 bead chips)	Not reported	2^*^	3^*^
Zhang et al. [8]	2008	TaqMan miRNA Assay (PE Applied biosystems, Foster City, CA)	35	4	31
Nam et al. [9]	2008	mirVana miRNA Probe Set (Ambion)	23	11	12
Iorio et al. [10]	2007	MiRNA microarray (Ohio State comprehensive cancer center, version 2.0)	29	4	25
Dahiya et al. [11]	2008	MiRCURY^TM^ LNA miRNA Arrays (Exiqon)	70	27	43
Yang et al. [12]	2008	Oligonucleotide arrays	14	7	7
Wyman et al. [13]	2009	454 Life sciences	58	37	21
Chao et al. [14]	2011	TaqMan miRNA Assay (PE Applied Biosystems, Foster City, CA)	17	15	2

* The authors did not showed the total number of the differently expressed miRNAs and they only selected two up-regulated and three down-regulated miRNAs for the follow-up experiments.

### Differentially expressed miRNAs

A total of 185 differentially expressed miRNAs were reported in the 8 miRNA expression profiling studies. The chromosomal localizations, pre-miRNA lengths, mature sequences, and the potential targets of the differentially expressed miRNAs are listed in the Tables 2, 3, 4 and 5.

**Table 2:** The differentially expressed miRNAs (n = 17) reported in at least three or more expression profiling studies

**miRNAs name**	**Chromosomal localization**	**Pre-miRNA length**	**Mature sequence**	**Potential target**	**References**
miR 200a	1p36.33	90 nt	54∣5′-UAACACUGUCUGGUAACGAUGU-3′∣75	ZEB1; ZEB2; CCNL2;	[9-14]
miR 100	11q24.1	80 nt	13∣5′-AACCCGUAGAUCCGAACUUGUG-3′∣34	TARDBP; FRAP1	[8-13]
miR 141	12p13.31	95 nt	17∣5′-CAUCUUCCAGUACAGUGUUGGA-3′∣38	AP3S1; ZEB1	[9-11,13,14]
miR 99a	21q21.1	81 nt	13∣5′-AACCCGUAGAUCCGAUCUUGUG-3′∣34	TARDBP; FRAP1	[8-11,13]
miR 200b	1p36.33	95 nt	57∣5′-UAAUACUGCCUGGUAAUGAUGAC-3′∣34	ZEB1; ZEB2; TARDBP;	[9,10,13,14]
miR 200c	12p13.31	68 nt	44∣5′-UAAUACUGCCGGGUAAUGAUGG-3′∣34	ZEB1; ZEB2; TARDBP;	[9,10,13,14]
miR 143	5q32.33	106 nt	61∣5′-UGAGAUGAAGCACUGUAGCUCA-3′∣82	ENO1; KIF1B; K-Ras	[9,10,13]
miR 145	5q32	88 nt	16∣5′-GUCCAGUUUUCCCAGGAAUCCCUU-3′∣39	RERE; CTNNBIP1; FSCN1	[8-10]
miR 214	1q23	110 nt	71∣5′-ACAGCAGGCACAGACAGGCAG-3′∣91	CCNL2; WDR8; WDR8	[9,10,12]
miR 134	q32.31	73 nt	8∣5′-UGUGACUGGUUGACCAGAGGGG-3′∣29	VEGFR; ABCC1	[9,10,12]
miR 154	14q32.31	84 nt	15∣5′-UAGGUUAUCCGUGUUGCCUUCG-3′∣36	FAM63B; GALNT7	[8,10,11]
miR 424	Xq26.3	98 nt	11∣5′-CAGCAGCAAUUCAUGUUUUGAA-3′∣32	FGF2	[8,11,14]
miR 29a	7q32.3	64 nt	42∣5′-UAGCACCAUCUGAAAUCGGUUA-3′∣63	C4orf32	[8,11,12]
miR 21	17q23.1	72 nt	8∣5′-UAGCUUAUCAGACUGAUGUUGA-3′∣29	CLCA3P; SATB1	[9,11,12]
miR 10b	2q31.1	110 nt	27∣5′-UACCCUGUAGAACCGAAUUUGU-3′∣34	CTNNBIP1; VPS13D	[9,11,13]
miR 26a	3p22.2 or 12q14.1	77 nt or 84 nt	10∣5′-UUCAAGUAAUCCAGGAUAGGC-3′∣30 or 14∣5′-UUCAAGUAAUCCAGGAUAGGC-3′∣34	UBE4B	[8,9,13]
Let 7d	9q22.32	87 nt	8∣5′-AGAGGUAGUAGGUUGCAUAGUU-3′∣29	HMGA2; C14orf28	[8,10,11]

**Table 3:** The differentially expressed miRNAs (n = 10) with an consistent direction between two studies

**miRNAs name**	**Chromosomal localization**	**Pre-miRNA length**	**Mature sequence**	**Direction of expression**	**Potential target**	**Reference**
miR 182	7q32.2	110 nt	23∣5′-UUUGGCAAUGGUAGAACUCACACU-3′∣46	↑	NRCAM; RGS17	[8]
				↑		[14]
miR 16	13q14.2	89 nt	14∣5′-UAGCAGCACGUAAAUAUUGGCG-3′∣35	↑	UNC80; KIF21A	[9]
				↑		[13]
miR 29c	1q32.2	88 nt	54∣5′-UAGCACCAUUUGAAAUCGGUUA-3′∣75	↑	COL3A1	[13]
				↑		[11]
miR 224	Xq28	81 nt	8∣5′-CAAGUCACUAGUGGUUCCGUU-3′∣28	↓	CDADC1; UBXN4	[8]
				↓		[10]
miR 125b	11q24.1	88 nt	15∣5′-UCCCUGAGACCCUAACUUGUGA-3′∣36	↓	CSNK2A1; FAM169B	[9]
				↓		[12]
miR 127	14q32.2	97 nt	23∣5′-CUGAAGCUCAGAGGGCUCUGAU-3′∣44	↓	NEK1	[8]
				↓		[10]
Let-7a	9q22.32	80 nt	6∣5′-UGAGGUAGUAGGUUGUAUAGUU-3′∣27	↓	C14orf28; HMGA2	[12]
				↓		[10]
Let-7c	21q21.1	84 nt	11∣5′-UGAGGUAGUAGGUUGUAUGGUU-3′∣32	↓	C14orf28; HMGA2	[10]
				↓		[12]
Let-7b	22q13.31	83 nt	6∣5′-UGAGGUAGUAGGUUGUGUGGUU −3′∣27	↓	C14orf28; HMGA2	[9]
				↓		[12]
miR 125a	19q13.41	86 nt	15∣5′-UCCCUGAGACCCUUUAACCUGUGA −3′∣38	↓	CSNK2A1; ZNF543	[9]
				↓		[10]

**Table 4:** The differentially expressed miRNAs (n = 7) with an inconsistent direction between two studies

**miRNAs name**	**Chromosomal localization**	**Pre-miRNA length**	**Mature sequence**	**Direction of expression**	**Potential target**	**Reference**
miR 494	14q32.31	81 nt	48∣5′-UGAAACAUACACGGGAAACCUC-3′∣69	↑	ARID4B; DZIP3	[11]
				↓		[12]
miR 221	Xp11.3	110 nt	65∣5′-AGCUACAUUGUCUGCUGGGUUUC-3′∣87	↑	LRRCC1; GABRA1	[11]
				↓		[13]
miR 519a	19q13.42	87 nt	54∣5′-AAAGUGCAUCCUUUUAGAGUGU-3′∣75	↑	FILIP1L	[11]
				↓		[7]
miR 335	7q32.2	94 nt	16∣5′-UCAAGAGCAAUAACGAAAAAUGU-3′∣38	↑	SMARCA2; LMX1A	[13]
				↓		[11]
miR 126	9q34.3	85 nt	52∣5′-UCGUACCGUGAGUAAUAAUGCG-3′∣73	↑	HOXA3; PPP3CB	[13]
				↓		[10]
miR 199a	19p13.2	71 nt	6∣5′-CCCAGUGUUCAGACUACCUGUUC-3′∣28	↑	ZNF763	[12]
				↓		[10]
miR 138	16q13	84 nt	10∣5′-AGCUGGUGUUGUGAAUCAGGCCG-3′∣32	↑	PKP4; RIMS2	[14]
				↓		[11]

**Table 5:** The differentially miRNAs (n = 5) with an consistent direction among four or more studies

**miRNAs name**	**Chromosomal localization**	**Pre-miRNA length**	**Mature sequence**	**Reference**	**Direction of expression**	**Potential target**
miR 200a	1p36.33	90 nt	54∣5′-UAACACUGUCUGGUAACGAUGU-3′∣75	[9]	↑	ZEB1; ZEB2;
				[10]	↑	CCNL2
				[12]	↑	
				[13]	↑	
				[14]	↑	
				[11]	↓	
miR 100	11q24.1	80 nt	13∣5′-AACCCGUAGAUCCGAACUUGUG-3′∣34	[11]	↑	TARDBP; FRAP1
				[12]	↓	
				[8]	↓	
				[10]	↓	
				[9]	↓	
				[13]	↓	
miR 141	12p13.31	95 nt	17∣5′-CAUCUUCCAGUACAGUGUUGGA-3′∣38	[13]	↑	AP3S1; ZEB1
				[9]	↑	
				[10]	↑	
				[14]	↑	
				[11]	↓	
miR 200b	1p36.33	95 nt	57∣5′-UAAUACUGCCUGGUAAUGAUGAC-3′∣34	[10]	↑	ZEB1; ZEB2;
				[14]	↑	TARDBP;
				[9]	↑	
				[13]	↑	
miR 200c	12p13.31	68 nt	44∣5′-UAAUACUGCCGGGUAAUGAUGG-3′∣34	[13]	↑	ZEB1; ZEB2;
				[14]	↑	TARDBP;
				[10]	↑	
				[9]	↑	

### Consistently reported, inconsistently reported and most consistently reported differentially expressed miRNAs

Among the 185 differentially expressed miRNAs reported from eight studies, 17 miRNAs were reported in at least three or more expression profiling studies; 6 (35.3%) with a consistent direction and 11 (64.7%) with an inconsistent direction among the studies (Table 2). MiR-200a and miR-100 were reported in 6 studies; MiR-141 and miR-99a were reported in 5 studies; MiR-200b and miR-200c were reported in 4 studies and 11 miRNAs (miR-143, miR-145, miR-214, miR-134, miR-154, miR-424, miR-29a, miR-21, miR-10b, miR-26a, and let-7d) were reported in 3 studies. Additionally, 10 miRNAs were reported with consistent direction between two studies (Table 3) and 7 miRNAs were reported with inconsistent direction between two studies (Table 4). More importantly, 5 differentially miRNAs (miR-200a, miR-100, miR-141, miR-200b, and miR-200c) were reported with the consistent direction among four or more studies (Table 5).

### Experimental validation of expression of the most consistently differentially expressed miRNAs in patients with EOC

**Figure 1:**
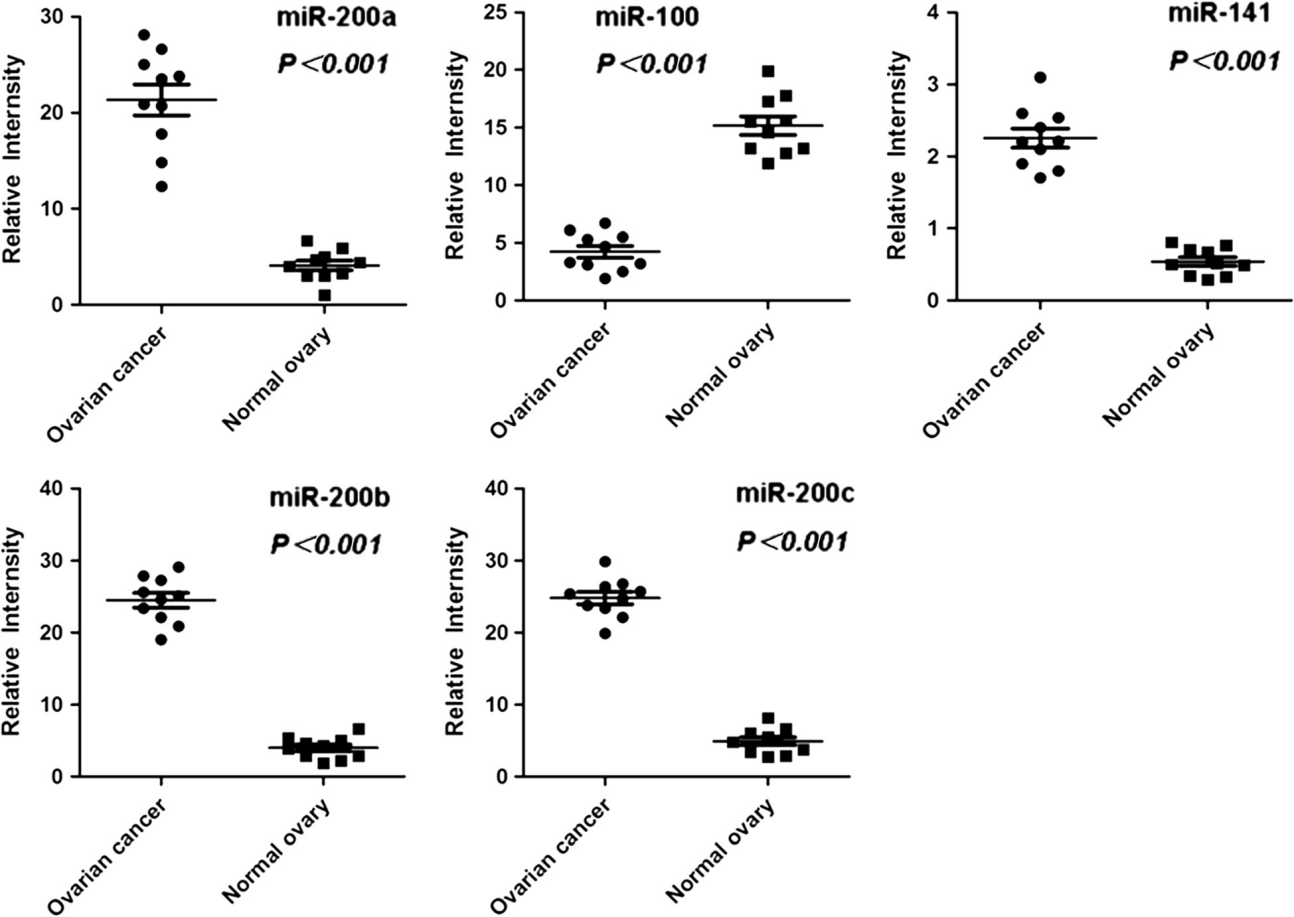
**qRT-PCR analysis of miR-200a, miR-100, miR-141, miR-200b, and miR-200c expressions in the EOC and normal ovarian tissues.** The results showed that the level of miR-100 was decreased, whereas the levels of miR-200a, miR-200b, miR-200c, and miR-141 were all increased in the EOC tissues, compared to the normal ovarian tissues (all of them *P<0.001*).

To validate the expression of the five most consistently reported miRNAs (miR-200a, miR-100, miR-141, miR-200b, and miR-200c), that may be the candidate biomarkers for EOC, the expression of these miRNAs between EOC and normal ovarian tissues were compared using qRT-PCR analysis. The preliminary results showed that the level of miR-100 was decreased, whereas the levels of miR-200a, miR-200b, miR-200c, and miR-141 were all increased in the EOC tissues, compared to the normal ovarian tissues (all of them *P<0.001*) (Figure. 1).

## Discussion

The common drawback of miRNA expression profiling studies is a lack of agreement among several studies. With reference to previous studies, a logical solution to the problem is to determine the agreement among the miRNA expression profiling studies using different platforms and observe which differentially expressed miRNAs are consistently reported. Notably, cumulative data had demonstrated that several consistently expressed miRNAs, maybe as candidate biomarker, were showed in some profiling studies of colorectal carcinoma [20] and lung cancer [21], respectively.

However, the general and accurate study of consistent miRNA expression in ovarian cancer versus normal ovary or benign ovarian tumors profiles had not been reported before. In this study, we observed that a total of 185 differentially expressed miRNAs were reported in the 8 miRNA expression profiling studies, but only 17 miRNAs were reported in at least three or more expression profiling studies. Excitingly, among the 17 miRNAs, 5 promising differentially miRNAs (miR-200a, miR-100, miR-141, miR-200b, and miR-200c) were reported with the consistent direction in four or more studies. It is that MiR-100 was down-regulated and the others all up-regulated in EOC tissues. Then, in order to determine whether the above five identified miRNAs had been previously validated to have diagnostic or prognostic values as biomarkers in EOC, a literature review and validation experiment were performed. Gratifyingly, the data of using EOC and normal ovarian specimens collected in our center was consistent with the findings of profiling studies.

Particularly, all of them (miR-200a, miR-200b, miR-200c, and miR-141), four up-regulated miRNAs of the five most consistently expressed miRNA among the eight profiling studies, belong to the miR-200 family. MiR-200a and miR-200b are located on chromosome 1, while miR-200c and miR-141 are on chromosome 12. The most prominent targets of the miR-200 family are two E-box binding transcription factors, ZEB1 and ZEB2, keys regulators of a complex network of transcriptional repressors regulating E-cadherin expression and epithelial polarity. Consistent with this function, the miR-200 family was recently identified as a marker, as well as a powerful regulator of the epithelial-to-mesenchymal transition (EMT). Indeed, EMT played an important role in carcinogenesis and tumor progression, when tumor cells undergo a change from a differentiated to a dedifferentiated, more aggressive and invasive phenotype. Furthermore, the increased expression of miR-200a might underlie genomic amplification. Frequent chromosomal gains in that region have been reported in ovarian carcinoma [22]. The inhibition of miR-200b increased the sensitivity to gemcitabine in cholangiocarcinoma cell lines [23], and thereby, increased expression of miR-200b may result in poor response to antineoplastic drugs. Moreover, inhibition of miR-141 using anti-miR-141 decreased cell growth in choloangiocarcinoma cell lines [23]. Accordingly, miR-200b and miR-141 could be a novel target for enhancing chemosensitivity or inducing cell death in ovarian cancer. Additionally, high expression of miR-200a was identified to correlate with decreased progression-free survival and overall survival for EOC patients significantly, even as miR-200c [9,16]. As a result, miR-200 family might serve as a molecular marker for the prediction of the prognosis and the evaluation of the response to chemotherapy for EOC patients.

MiR-100, another important consistent expressed miRNA in this study, may be as another candidate biomarker in ovarian cancer, which has been reported to be down-regulated in 5 profiling studies but up-regulated only in one study. MiR-100 represses mTOR (mammalian target of rapamycin) signaling and increases sensitivity to the cancer drug everolimus (rapamycin analog RADOO1) in cell lines derived from clear cell carcinomas [24]. mTOR is a serine/threonine kinase and downstream effector of the AKT signaling pathway, which has also been shown to be a possible therapeutic target in both cisplatin-sensitive and cisplatin-resistant clear cell ovarian carcinoma [24,25]. Additionally, miR-100 inhibited cell proliferation by suppressing mTOR in esophageal squamous cell carcinoma (ESCC) cell lines and Low miR-100 expression was associated with worse overall survival in ESCC patients [26]. Recently, Peng et al. showed that miR-100 can significantly inhibit growth of EOC cells by targeting PLK1 (Polo-like kinase-1) and more importantly, miR-100 may be as an independent predictor for the prognosis evaluation of ovarian cancer patients. Thus, they suggested the miR-100/PLK1 signaling pathway may provide therapeutic targets for human EOCs [17].

Conclusively, this systematic review and validation experiment demonstrated four up-regulated miRNAs (miR-200a, miR-200b, miR-200c and miR-141) and one down-regulated miRNA (miR-100) are promising important candidate biomarkers for EOC. However, the shortcomings of this study are review of profiling studies with retrospective and limited profiles. Further clinical and mechanistic studies focusing on these miRNA need to be performed for their clinical significance and the underlying roles in tumorigenesis of EOC.

## Competing interest

None of the authors has any potential conflicts of interest.

## Authors’ contributions

YC, LZ and QH summarized and analyzed the data. YC designed the experiments and wrote the paper. All authors have read and approved the final manuscript.
